# Effects of donepezil 23 mg on Severe Impairment Battery domains in patients with moderate to severe Alzheimer's disease: evaluating the impact of baseline severity

**DOI:** 10.1186/alzrt166

**Published:** 2013-02-21

**Authors:** Steven Ferris, Jeffrey Cummings, Daniel Christensen, Rachelle Doody, Martin Farlow, Marwan Sabbagh, Liang Liu, Joan Mackell, Randi Fain

**Affiliations:** 1 Alzheimer Disease Center, New York University Langone Medical Center, 145 E 32nd St, Room 506, New York, NY 10016, USA; 2 Cleveland Clinic Lou Ruvo Center for Brain Health, 888 W. Bonneville, Las Vegas, NV 89106, USA; 3 Neuropsychiatric Institute, University of Utah, 501 Chipeta Way, Salt Lake City, UT 84108, USA; 4 Department of Neurology, Alzheimer's Disease and Memory Disorders Center, Baylor College of Medicine, Houston, TX 07730, USA; 5 Department of Neurology, Indiana University School of Medicine, 541 Clinical Drive, CL299, Indianapolis, IN, 46202, USA; 6 The Cleo Roberts Center for Clinical Research, Banner-Sun Health Research Institute, 10515 W. Santa Fe Drive, Sun City, AZ 85351, USA; 7 Eisai Inc., 100 Tice Boulevard, Woodcliff Lake, NJ 07677, USA; 8 Pfizer Inc, 235 East 42nd Street, New York, NY 10017, USA

## Abstract

**Introduction:**

The US Food and Drug Administration approved a 23 mg daily dose of donepezil for treatment of moderate to severe Alzheimer's disease (AD) based on outcomes from a large trial comparing the 23 mg/day dose with the standard 10 mg/day dose. Results from this study indicated that after 24 weeks, donepezil 23 mg/day provided significant cognitive benefits over donepezil 10 mg/day, measured using the Severe Impairment Battery (SIB). In the analyses reported herein, we further characterize the range of cognitive domains impacted by treatment with donepezil 23 mg/day.

**Methods:**

A post hoc analysis was conducted using data from a 24-week, randomized, double-blind trial comparing donepezil 23 mg/day versus 10 mg/day in 1,467 patients with moderate to severe AD (baseline Mini-Mental State Examination (MMSE) score 0 to 20). Changes from baseline to week 24 in the nine SIB domain scores were analyzed in the intent-to-treat (ITT) population (baseline MMSE 0 to 20), in patients with more severe baseline AD (MMSE 0 to 16), and in severity strata based on baseline MMSE scores (0 to 5, 6 to 10, 11 to 15, 16 to 20).

**Results:**

In the ITT population, changes in six of the nine SIB domains favored donepezil 23 mg/day over donepezil 10 mg/day. LS mean treatment differences were significant for the language, visuospatial ability, and construction domains. In the more advanced cohort of patients (MMSE 0 to 16 at baseline), LS mean treatment differences were statistically significant favoring donepezil 23 mg/day in five of the nine domains: language, memory, visuospatial ability, attention, and construction. Descriptive analysis of LS mean changes in SIB domain scores in the four baseline severity strata showed variable patterns of response; overall, cognitive benefits of donepezil 23 mg/day were greatest in patients with MMSE scores of 0 to 15.

**Conclusions:**

These results suggest that donepezil 23 mg/day provides benefits over 10 mg/day across a range of cognitive domains. The magnitude of benefit and domains impacted varied depending on the stage of AD; significant benefits with higher dose donepezil were most apparent at more advanced stages of AD and were most prominent in the language domain.

## Introduction

Alzheimer's disease (AD) is a progressive neurodegenerative disorder affecting more than 5 million people in the United States and 35 million people worldwide [1]. Based on the latest estimates, more than half of these individuals would be classified as having moderate or severe AD [2]. These more advanced stages of the disease can last for up to a decade [3,4], placing a great burden on families and caregivers and resulting in substantial health care costs [1].

Cognitive dysfunction in the early stages of AD has been well studied using clinical trial assessment scales such as the Alzheimer's Disease Assessment Scale (ADAS) [5,6], a valid and reliable tool for evaluating cognition in patients at less advanced stages of the disease; however, when patients progress to moderate to severe stages of AD, this scale is no longer effective or reliable. Floor effects, in particular, limit its usefulness in measuring cognitive capabilities or changes in cognitive function related to therapy [7]. The Severe Impairment Battery (SIB) was originally developed more than 20 years ago to assess cognition in patients with more advanced AD [8-11]. It uses one-step conversational commands presented with gestural cues, which enhances its utility in patients with profound impairment in their ability to communicate. The SIB is a reliable and sensitive assessment tool when used in patients progressing from moderate to severe AD. It has been widely used and validated as a measure of cognitive function in several clinical trials [3,7,12-15]. The SIB was designed to assess cognitive function across nine domains: language, memory, praxis, visuospatial ability, attention, orientation, social interaction, construction, and orienting to name [8].

Donepezil, a selective, reversible acetylcholinesterase inhibitor, is approved in the US for the treatment of AD in the moderate to severe stages. In addition to standard 5 mg and 10 mg daily doses, the US Food and Drug Administration (FDA) has approved the use of a higher daily dose of donepezil (23 mg) to treat patients with moderate to severe AD [16]. Approval of the 23 mg daily dose was based on outcomes from a large, randomized clinical trial comparing donepezil 23 mg/day with donepezil 10 mg/day [17]. Results from this study indicated that after 24 weeks of treatment donepezil 23 mg/day provided statistically significant incremental cognitive benefits over donepezil 10 mg/day, as measured using the SIB. For the global function co-primary end point (Clinician's Interview-Based Impression of Change-plus caregiver input (CIBIC-plus)), no statistically significant incremental benefits of donepezil 23 mg/day over 10 mg/day were observed. A subsequent post hoc analysis, focused on evaluating the possible differential effects of the two donepezil doses on language function, indicated that the overall cognitive benefits of donepezil 23 mg/day over 10 mg/day were driven at least in part, by language benefits [18]. Herein we report the results of additional post hoc analyses of data from the trial of donepezil 23 mg/day versus donepezil 10 mg/day to further characterize the range of cognitive domains impacted by treatment with donepezil 23 mg/day. In order to investigate treatment responses across a spectrum of disease severity, we studied effects in the overall intent-to-treat (ITT) population (all Mini-Mental State Examination (MMSE) scores 0 to 20), in a cohort of patients with more severe baseline impairment (MMSE 0 to 16 at baseline) as determined in the original study report [17], and in further severity strata (MMSE 0 to 5, 6 to 10, 11 to 15, and 16 to 20).

## Materials and methods

### Study design

Methods used in the original clinical trial have been described in detail previously [17]. In brief, this was a 24-week, double-blind, parallel group, global study including 1,467 patients aged 45 to 90 years, who were diagnosed with moderate to severe AD (baseline MMSE scores 0 to 20) and who had been receiving a stable dose of donepezil 10 mg/day for at least 3 months. Patients were randomized in a ratio of 2:1 to increase their daily dose of donepezil to 23 mg/day, or to continue taking their existing dose of 10 mg/day. The pre-specified co-primary efficacy measures from the original study were change from baseline in SIB total score (cognition) and the CIBIC-plus global change scores at week 24. The protocol and informed consent form for the original trial were approved by the independent ethics committee and institutional review board of each independent research site and conformed to the principles of the World Medical Association Declaration of Helsinki and all local regulations. Written informed consent was obtained from each patient where possible, or from the patient's legal guardian or representative. If a patient was unable to provide written consent, he or she was required to provide verbal agreement to participate in the study, with documentation of this assent noted in the study record [17].

### SIB scale

**Figure 1:**
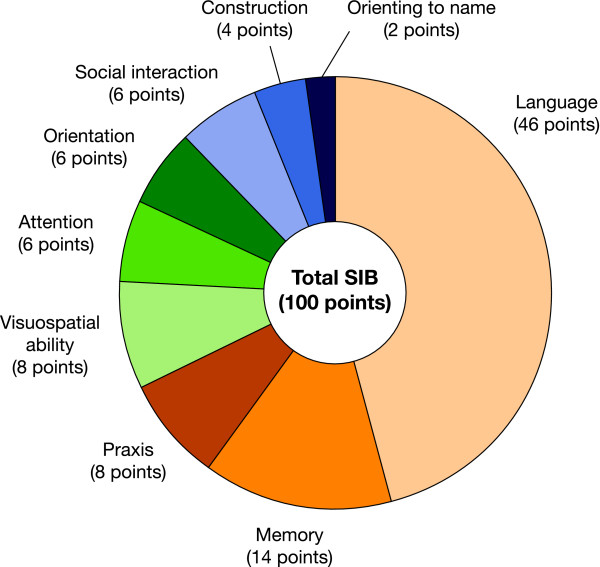
**Severe Impairment Battery domains and scale points allocated**. SIB, Severe Impairment Battery.

The SIB includes 40 items divided across the nine cognitive domains (Figure 1) [8,10]. The subdomains of the SIB are weighted differently, with language making up the largest proportion (Figure 1). The total SIB score for each patient consists of the sum of all responses on the nine SIB domains and ranges from 0 (worst score) to 100 (best score). SIB assessments were performed at screening, baseline, and weeks 6, 12, 18, and 24.

### Analyses

Post hoc analysis of changes from baseline to week 24 in individual SIB domain scores was performed in the overall ITT population (all MMSE scores 0 to 20), in the cohort of patients with more severe baseline impairment (MMSE 0 to 16 at baseline), and in further severity strata based on baseline MMSE (0 to 5, 6 to 10, 11 to 15, and 16 to 20).

Analysis of changes in SIB domain scores at week 24 was performed using an analysis of covariance model with country, treatment, and baseline as interaction terms. For efficacy analysis in the patient populations and subpopulations, missing SIB values were imputed by the last observation carried forward (LOCF) method. Standardized effect sizes for the nine SIB domains were calculated for the overall patient population and subpopulations (overall ITT, MMSE 0 to 16, MMSE 0 to 5, MMSE 6 to 10, MMSE 11 to 15, MMSE 16 to 20) by dividing the treatment differences of least squares (LS) means by the pooled SDs. A positive effect size means that on average donepezil 23 mg performed better than donepezil 10 mg, while the reverse is true for a negative effect size. For positive effect sizes, the higher the effect size, the greater the difference in treatment effect between the donepezil 23 mg and donepezil 10 mg groups. The pooled SD is the SD of change from baseline of SIB scores from all subjects, regardless of actual treatment.

## Results

### Patients

In total, 1,467 patients were randomized to donepezil 23 mg/day (*n *= 981) and donepezil 10 mg/day (*n *= 486) in the original trial. Of these, 1,371 patients (909 patients receiving donepezil 23 mg/day and 462 receiving donepezil 10 mg/day) comprised the overall ITT population. Patient baseline demographics and clinical characteristics were similar for both donepezil treatment groups, as has been previously reported [17,18]. Relevant baseline demographics and characteristics are shown for the ITT population in Table 1. Baseline disease severity, as assessed using the MMSE and SIB, was comparable between the two treatment groups. There were no notable differences in individual SIB domain baseline scores (MMSE 0 to 20) (Table 1).

**Table 1:** Demographics and baseline characteristics (intent-to-treat population).

Characteristics	Donepezil 10 mg/day	Donepezil 23 mg/day
Age		
Mean (SD)	73.8 (8.6)	73.8 (8.5)
	(*n *= 462)	(*n *= 909)
Gender		
Male, n (%)	175 (37.9)	335 (36.9)
Female, n (%)	287 (62.1)	574 (63.1)
Total patients, n	462	909
ADCS-ADL-sev		
Mean (SD)	34.5 (11.2)	34.1 (10.9)
	(*n *= 461)	(*n *= 908)
CIBIS-plus Mean (SD)	4.38 (0.89)	4.42 (0.85)
	(*n *= 461)	(*n *= 904)
MMSE		
Mean (SD)	13.1 (4.7)	13.1 (4.99)
	(*n *= 462)	(*n *= 908)
SIB		
Mean (SD)	75.6 (16.3)	74.2 (17.6)
	(*n *= 462)	(*n *= 907)
Individual SIB domain scores, mean (SD):		
Language	36.7 (8.40)	36.2 (9.02)
Memory	8.5 (3.03)	8.2 (3.24)
Praxis	5.5 (2.33)	5.4 (2.45)
Visuospatial ability	6.5 (2.04)	6.3 (2.34)
Attention	4.3 (1.65)	4.2 (1.66)
Orientation	3.7 (1.45)	3.7 (1.45)
Social	5.3 (1.14)	5.3 (1.06)
Construction	3.4 (1.16)	3.3 (1.25)
Orienting to name	1.7 (0.59)	1.6 (0.60)
Total patients, n	462	907

ADCS-ADL-sev, Alzheimer's Disease Cooperative Study-Activities of Daily Living Inventory, severe version; CIBIS-plus, Clinician's Interview-Based Impression of Severity-plus caregiver input; MMSE, Mini-Mental State Examination; SIB, Severe Impairment Battery; SD, standard deviation.

### Analyses: treatment effects on individual domains of the SIB scale

#### Treatment effects in the overall ITT population (MMSE 0 to 20)

**Figure 2:**
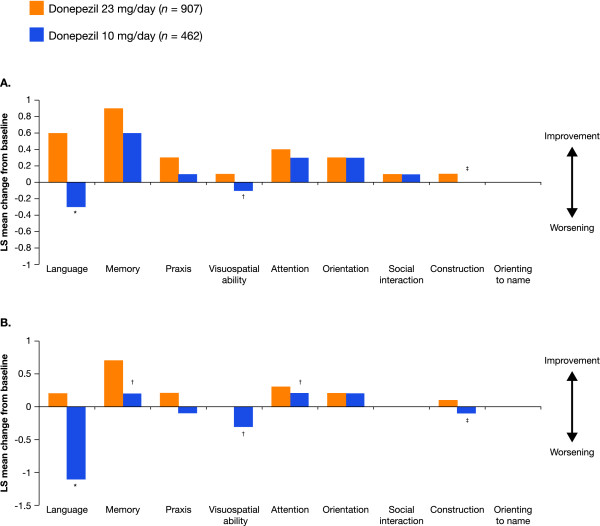
**Change from baseline in Severe Impairment Battery subdomain scores at week 24 (last observation carried forward)**. *P <0.001, ^†^*P *<0.05, ^‡^*P *<0.01. (**A**) Overall ITT population (MMSE 0 to 20). (**B**). MMSE 0 to 16. ITT, intent to treat; MMSE, Mini-Mental State Examination; LS, least squares.

For the overall ITT population, scores in eight of the nine individual SIB domains improved from baseline with donepezil 23 mg/day, the exception being orienting to name scores, which were relatively unchanged (Figure 2). No decline from baseline was seen in individual SIB domain scores among the patients receiving donepezil 23 mg/day. In the donepezil 10 mg/day treatment group, scores on five of the SIB domains (memory, praxis, attention, orientation, and social interaction) were improved from baseline, scores on two (construction and orienting to name) were stable, and scores on two (language and visuospatial ability) declined. Changes in SIB scores numerically favored patients treated with donepezil 23 mg/day over donepezil 10 mg/day for six of the nine SIB domains (language, memory, praxis, visuospatial ability, attention, and construction). These treatment differences were statistically significant in favor of donepezil 23 mg/day for three domains (language (*P *<0.001), visuospatial ability (*P *<0.05), and construction (*P *<0.01)).

Larger effect sizes indicating greater benefits with donepezil 23 mg/day over donepezil 10 mg/day were observed for the language, construction, visuospatial ability, praxis, memory, and attention domains (Table 2); these domains encompass the majority of items on the SIB. The greatest effect in favor of 23 mg was seen in the language domain (effect size = 0.191), which comprises 46 of 100 points on the total SIB scale. Treatment differences and effect sizes were negligible for the orientation, social interaction, and orienting to name domains.

**Table 2:** Standardized effect sizes by individual SIB domains for the overall study population and patient subgroups (intent-to-treat population, last observation carried forward)

Severe Impairment Battery (SIB) domain		Mini-Mental State Examination score					
		
		0 to 20	0 to 16	0 to 5	6 to 10	11 to 15	16 to 20
Language	LS mean treatment difference	0.903	1.310 (0.63 to	2.602	1.686	0.691	0.141
	(95% CI)*	(0.38 to 1.43)	1.99)	(0.17 to 5.04)	(0.25 to 3.12)	(-0.19 to 1.57)	(-0.40 to 0.68)
	Standardized effect size^†^		0.253 (5.171)			0.152	0.045
	(pooled SD)	0.191		0.396	0.289	(4.558)	(3.150)
		(4.736)		(6.571)	(5.828)		
Memory	LS mean treatment difference	0.269	0.421	0.871 (-0.19	0.249 (-0.44	0.237 (-0.22 to	0.087 (-0.32 to
	(95% CI)*	(-0.02 to 0.56)	(0.07 to 0.77)	to 1.93)	to 0.94)	0.69)	0.50)
	Standardized effect size^†^		0.155 (2.711)	0.304 (2.863)	0.086 (2.893)	0.093 (2.534)	0.035 (2.469)
	(pooled SD)	0.101					
		(2.675)					
Praxis	LS mean treatment difference	0.216	0.252 (-0.02 to	0.854 (0.10 to	0.238 (-0.34	0.099 (-0.27 to	0.108 (-0.16 to
	(95% CI)*	(-0.00 to 0.43)	0.52)0.115	1.61)0.415	to 0.81)0.099	0.47)0.046	0.37)0.061
	Standardized effect size^†^		(2.192)	(2.060)	(2.417)	(2.148)	(1.753)
	(pooled SD)	0.104					
		(2.077)					
Visuospatial ability	LS mean treatment difference	0.195	0.311	0.576 (-0.36	0.506 (-0.04	-0.004 (-0.30	0.049 (-0.16 to
	(95% CI)*	(0.00 to 0.39)	(0.06 to 0.56)	to 1.51)0.221	to 1.05)0.230	to 0.29)-0.002	0.26)
	Standardized effect size^†^		0.161 (1.934)	(2.603)	(2.198)	(1.634)	0.039 (1.284)
	(pooled SD)	0.110					
		(1.772)					
Attention	LS mean treatment difference	0.123	0.179	0.211 (-0.33	0.098 (-0.26	0.240 (0.02 to	0.032 (-0.17 to
	(95% CI)*	(-0.02 to	(0.01 to 0.35)	to 0.76)0.141	to 0.46)0.064	0.46)0.195	0.23)0.026
	Standardized effect size	0.26)0.093	0.132	(1.500)	(1.516)	(1.233)	(1.231)
	(pooled SD)	(1.326)	(1.357)				
Orientation	LS mean treatment difference	0.038 (-0.10	0.056 (-0.11 to	-0.028 (-0.45	-0.003 (-0.31	0.152 (-0.08 to	-0.033 (-0.24
	(95% CI)^*^	to 0.18)0.028	0.22)0.041	to 0.40)-0.024	to 0.30)-0.002	0.38)0.105	to 0.18)-0.025
	Standardized effect size^†^	(1.354)	(1.367)	(1.160)	(1.352)	(1.445)	(1.290)
	(pooled SD)						
Social interaction	LS mean treatment difference	-0.003 (-0.10	0.028	0.423 (-0.16	-0.077 (-0.35	-0.071 (-0.22	-0.064 (-0.18
	(95% CI)*	to 0.10)-0.003	(-0.10 to	to 1.00)0.273	to 0.20)-0.065	to 0.08)-0.075	to 0.05)-0.084
	Standardized effect size^†^	(1.012)	0.16)0.026	(1.547)	(1.189)	(0.956)	(0.762)
	(pooled SD)		(1.086)				
Construction	LS mean treatment difference	0.139 (0.04 to	0.176 (0.04 to	0.505 (0.04 to	0.220 (-0.10	0.053 (-0.10 to	0.062 (-0.04 to
	(95% CI)*	0.24)0.146	0.31)0.168	0.97)0.395	to 0.54)0.166	0.21)0.061	0.16)0.097
	Standardized effect size^†^	(0.951)	(1.045)	(1.280)	(1.324)	(0.863)	(0.642)
	(pooled SD						
Orienting to name	LS mean treatment difference	-0.026 (-0.08	-0.024 (-0.10 to	0.050 (-0.22	-0.043 (-0.18	-0.046 (-0.14	-0.023 (-0.10
	(95% CI)*	to 0.03)-0.042	0.05)-0.037	to 0.32)0.065	to 0.10)-0.063	to 0.05)-0.080	to 0.05)-0.038
	Standardized effect size^†^	(0.635)	(0.642)	(0.771)	(0.690)	(0.579)	(0.601)
	(pooled SD)						
Total SIB	LS mean treatment difference	2.152 (1.06 to	3.141 (1.74 to	6.048 (1.04	3.258 (0.30 to	1.697 (-0.11 to	0.504 (-0.67 to
	(95% CI)	3.24)0.218	4.54)0.296	to11.05)0.460	6.21)0.273	3.51)0.181	1.68)0.076
	Standardized effect size^†^	(9.864)	(10.619)	(13.150)	(11.941)	(9.376)	(6.657)
	(pooled SD)						

*Difference between donepezil 23 mg/day and 10 mg/day treatment groups for the score change from baseline to week 24; ^†^positive standardized effect sizes represent superiority of donepezil 23 mg/day dose over 10 mg/day dose. LS, least squares.

#### Treatment effects in the cohort with more severe baseline impairment (MMSE 0 to 16)

In the original clinical trial [17], post hoc sensitivity analyses of the impact of baseline disease severity on treatment response were conducted in a cohort of patients with baseline MMSE scores from 0 to 16 (more severe impairment). In the current study, in the same severity strata, improvement from baseline with donepezil 23 mg/day was demonstrated in six of the nine individual SIB domain scores (language, memory, praxis, attention, orientation, and construction), whereas scores for social interaction, visuospatial ability, and orienting to name were stable (Figure 2). No decline from baseline in individual SIB domain scores was seen among the patients receiving donepezil 23 mg/day. Patients treated with donepezil 10 mg/day had improved scores from baseline in three domains (memory, attention, and orientation), scores on two were stable (social interaction and orienting to name), and scores on four declined (language, praxis, visuospatial ability, and construction). In this more severely impaired cohort, changes in five of the nine SIB domains comparing donepezil 23 mg/day to donepezil 10 mg/day were statistically significant (language (*P *<0.001), memory (*P *<0.05), visuospatial ability (*P *<0.05), attention (*P *<0.05), and construction (*P *<0.01)).

In this cohort of patients with more severe baseline impairment, the largest treatment effect was seen in the language domain (effect size = 0.253) (Table 2). Effect sizes also demonstrated similar cognitive benefits of donepezil 23 mg over 10 mg in the domains of construction, visuospatial ability, memory, attention, and praxis. As was seen in the overall patient population, treatment differences and effect sizes were negligible for the orientation, social interaction, and orienting to name domains. In all domains except orienting to name, effect sizes were greater in this cohort versus the overall ITT population.

#### Treatment effects in baseline severity strata

**Figure 3:**
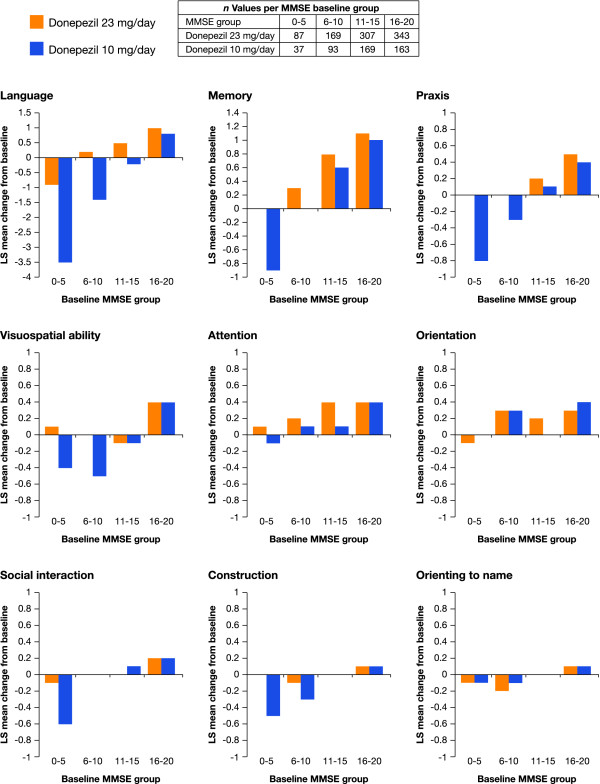
**Severe Impairment Battery domain scores - change from baseline to week 24, stratified by baseline Mini-Mental State Examination subgroups (intent-to-treat populations, last observation carried forward)**. MMSE, Mini-Mental State Examination; LS, least squares.

The LS mean changes in SIB domain scores from baseline to end point were also evaluated descriptively for each of the four baseline severity strata (Figure 3). This subgroup analysis revealed variable patterns of response. Patients in each of the two more severe strata (MMSE 0 to 5 and 6 to 10) tended to exhibit the greatest treatment differences in favor of donepezil 23 mg/day for the domains of social interaction, memory, language, praxis, visuospatial ability, and construction. Patients in the two moderate strata (MMSE 11 to 15 and 16 to 20) tended to show smaller between-treatment differences, and in some domains (visuospatial ability, construction, and orienting to name) no between-treatment differences were seen in these strata. Overall, the descriptive analysis across the four severity strata indicated that the cognitive benefits of donepezil 23 mg/day compared with donepezil 10 mg/day were greatest in patients with MMSE scores of 0 to 15.

The domains most positively impacted by treatment with donepezil 23 mg/day for the most severely impaired patient stratum (MMSE 0 to 5) were the praxis, language, construction, and memory domains (Table 2); treatment benefits to a lesser degree were also seen in the social interaction, visuospatial ability, and attention domains. Effect sizes were minimal in the orientation and orienting to name domains. For the MMSE 6 to 10 stratum, treatment effects were greatest in the language, visuospatial ability, and construction domains but relatively small for the remaining domains. The effect size data generally showed smaller differences in treatment effect between the 23 mg/day and 10 mg/day groups for the MMSE 11 to 15 and 16 to 20 patient strata. In the MMSE 11 to 15 group, the greatest effects were in the language, attention, and orientation domains, with lesser effects on memory, praxis, and construction, and no effect on the remaining domains. For the more moderately impaired patient stratum (MMSE 16 to 20), all the domains except orientation, social interaction, and orienting to name were minimally but positively affected by donepezil 23 mg/day treatment.

## Discussion

The current post hoc analysis was conducted to further characterize the nature of the cognitive benefits seen in the primary study of donepezil 23 mg/day versus donepezil 10 mg/day [17], and to assess the range of patients experiencing benefits from a transition from donepezil 10 mg/day to 23 mg/day. In the full study population, statistically significant incremental benefits of donepezil 23 mg/day over donepezil 10 mg/day were observed for the domains of language, visuospatial ability, and construction (MMSE 0 to 20). In the cohort of patients with more severe baseline disease (MMSE 0 to 16), significant clinical treatment benefits in favor of donepezil 23 mg/day were again evident in the domains of language, visuospatial ability, and construction, but also in the domains of memory and attention. Across both populations, scores declined with 10 mg on the domains of language, praxis, visuospatial ability, and construction, but scores on these domains were either improved or stabilized with 23 mg. Moreover, based on the standardized effect sizes analysis, the benefits of 23 mg over 10 mg were greater in the 0 to 16 cohort than the overall ITT population for most domains, further indicating that the transition from 10 to 23 mg may be most beneficial at advanced stages of AD. Donepezil 23 mg provided benefits over donepezil 10 mg on the majority of SIB domain scores across the four baseline severity strata. These benefits were numerically greatest in the patients with the most severe disease at baseline (MMSE 0 to 5) for social interaction, memory, language, praxis, visuospatial ability, and construction. Overall, most incremental benefits of treatment with donepezil 23 mg/day over 10 mg/day were seen in patients with more advanced baseline disease (MMSE 0 to 15). These findings suggest that despite the advancing cholinergic deficit in AD, increasing levels of cholinesterase inhibition may help maintain certain cognitive abilities as the disease progresses [19]. It may be the case that while patients with early-stage AD achieve sufficient response from lower-dose acetylcholinesterase inhibitors, patients with more advanced AD require higher doses to achieve an optimal response.

The investigation of subdomains on the SIB scale and the identification of specific areas of treatment effects have important clinical implications. The calculated standardized effect sizes demonstrated that donepezil 23 mg/day showed substantial benefits over 10 mg/day on a broad range of cognitive domains, specifically, language, construction, visuospatial ability, praxis, memory, and attention, and these benefits were evident in the overall moderate to severe study population (MMSE 0 to 20) as well as the patient cohort with more severe baseline disease (MMSE 0 to 16). However, for the most severely impaired patient group (MMSE 0 to 5), the greatest benefits of comparable magnitude were seen in the domains of praxis, language, and construction; a notable effect on memory and social interaction was also observed. Patients in the more moderate MMSE subgroups had smaller treatment effect sizes generally. These results suggest that treatment with donepezil 23 mg/day may help preserve patients' ability to communicate verbally as they move from moderate to more severe disease. Language improvements, together with improvements in construction and visuospatial ability associated with the donepezil 23 mg/day dose, may translate into everyday benefits for patients with AD in their ability to communicate and perform activities of daily living.

Previous studies have examined the impact of drug therapy on SIB domain scores in patients with moderate to severe AD. Pooled data from four clinical trials involving patients with moderate or severe AD treated with donepezil 5 mg/day or 10 mg/day or placebo (*n *= 904; MMSE 1 to 17) demonstrated that donepezil treatment provided significant improvement compared with placebo on seven of the nine SIB domain scores (excluding social interaction and orienting to name) [20]. The observation that the greatest benefit of donepezil 10 mg/day over placebo was in language, praxis, memory, and visuospatial ability domains was supported by the results of our study; data for the MMSE 0 to 16 population show that the greatest benefits of donepezil 23 mg/day over 10 mg/day were seen in the language, visuospatial ability, memory, and construction domains. Treatment benefit was seen in the praxis domain but to a lesser degree. Furthermore, in the pooled analysis by Cummings and colleagues [20], scores on almost all subdomains were improved from baseline to month 6 with donepezil 10 mg/day among the MMSE 1 to 5 and 6 to 9 groups. However, in the current study, scores on the same domains declined in the MMSE 0 to 5 and 6 to 10 groups with donepezil 10 mg/day (with the exception of attention and orientation). A factor contributing to this difference is that donepezil was initiated at the start of the donepezil 10 mg/day versus placebo study, whereas patients in the current study had been receiving donepezil 10 mg for a minimum of 3 months (with a mean duration of 2 years on donepezil 10 mg) at study initiation. Therefore, patients in the current study might be less likely to experience a further increase in symptomatic benefits from continued donepezil 10 mg/day treatment.

A study of galantamine therapy in patients with severe AD (baseline MMSE scores of 5 to 12; *n *= 407) demonstrated improvement in several SIB domains, with significant treatment benefit compared with placebo for the domains of memory (*P *= 0.006), praxis (*P *= 0.010), and visuospatial ability (*P *= 0.002) [21]. Although treatment effect sizes were not provided in this study, the greatest numerical benefits of galantamine over placebo seemed to be in the domains of language, praxis, memory, and visuospatial ability, as was the case in the study by Cummings and colleagues [20]. Studies of memantine treatment have also shown significant benefits compared with placebo for the SIB subdomains [12,13,22]. A post hoc analysis of data from six randomized, double-blind, 6-month studies of patients with moderate to severe AD showed a significant benefit of memantine treatment, compared with placebo, for the SIB subdomains of language (*P *<0.05), memory (*P *<0.05), orientation (*P *<0.01), praxis (*P *<0.001), and visuospatial ability (*P *<0.01) [22]. These studies demonstrate that patients with moderate to severe AD can be assisted with a variety of types of pharmacotherapy. There are differences between these prior studies and our study. Most importantly, the previous studies reported the efficacy of active drug versus placebo, whereas our results compare two doses of active drug. This may have affected comparisons as patients receiving placebo may decline more rapidly than those taking 10 mg/day donepezil. Second, effect size data are often not available from these prior studies.

Although the current analysis was designed to further investigate cognitive outcomes with donepezil 23 mg/day, the pivotal study that provided the data for this analysis also assessed the safety and tolerability of donepezil 23 mg/day compared with 10 mg/day over 24 weeks [17]. Subjects transitioning to higher-dose donepezil experienced an increase in gastrointestinal events, with more nausea and vomiting. These side effects occurred more frequently during the first month after starting treatment and, for patients who adhered to treatment, were relatively infrequent thereafter. Clinical benefits of donepezil 23 mg/day, in terms of cognitive changes such as those discussed herein, should be carefully assessed on a patient-by-patient basis together with the related side effect profile, to allow physicians to determine whether patients are benefiting from the higher dose of donepezil.

The limitations of this post hoc study should be considered when interpreting the results. Data for the LS mean treatment difference should be interpreted with caution, as the number of total possible points varies by SIB domain. The study was not powered for differences in subdomains and for this reason the statistical significance may be secondary to the consistent pattern of the findings.

## Conclusions

The results of this post hoc analysis suggest that donepezil 23 mg/day provides benefits over 10 mg/day across a range of cognitive domains in patients with moderate to severe AD. Although the magnitude of these benefits and the specific domains that were impacted varied depending on the severity of AD, the analyses indicated that significant incremental benefits with the higher donepezil dose (23 mg/day) were most prominent in the language domain and most apparent in patients at more advanced stages of the disease.

## Abbreviations

AD: Alzheimer's disease; ADAS: Alzheimer's Disease Assessment Scale; CIBIC-plus: Clinician's Interview-Based Impression of Change-plus caregiver input; FDA: US Food and Drug Administration; ITT: intent-to-treat; LOCF: last observation carried forward; LS: least squares; MMSE: Mini-Mental State Examination; SIB: Severe Impairment Battery.

## Competing interests

SF has served as a paid scientific consultant to Accera, Baxter, Bristol-Myers Squibb, Eisai, Elan, Eli Lilly, Janssen AI, Lupin, MedAvante, Merck, Merz, Neuronix, Novartis, Otsuka, Pfizer, Toyama, and United Biosource. His institution has received grant/contract support for clinical trials from Baxter, Bristol Myers-Squibb, Eisai, Eli Lilly, Janssen AI, Merck, Neuronix, Pfizer, Roche, and Takeda. He also has stock options from Accera, Intellect Neurosciences, MedAvante Neuronix, and Raptor, and owns stock in Lexicon. JC has provided consultation to Abbott, Acadia, ADAMAS, Anavex, Astellas, AstraZeneca, Avanir, Baxter, Bristol-Myers Squibb, Eisai, Elan, EnVivo, Forest, Genentech, GlaxoSmithKline, Janssen, Lilly, Lundbeck, Medtronics, Merck, Neurokos, Neuronix, Novartis, Otsuka, Pain Therapeutics, Pfizer, Plexxicon, Prana, QR, Sanofi, Sonexa, Takeda, and Toyama pharmaceutical companies. He has also provided consultation on diagnostic assessment to Bayer, Avid, GE, MedAvante, Neurotrax, and UBC. Dr. Cummings owns stock in ADAMAS, Prana, Sonexa, MedAvante, Neurotrax, Neurokos, and QR pharma. He is a speaker/lecturer for Eisai, Forest, Janssen, Novartis, Pfizer, and Lundbeck. Dr. Cummings owns the copyright of the Neuropsychiatric Inventory, and has provided expert witness consultation regarding olanzapine and ropinirole. DC has received consultant fees from Pfizer, Eisai, and Medivation, and has been on the Speakers' Bureau for Pfizer and Eisai; he holds stock in Pfizer, Eli Lily, BMS and Johnson & Johnson. RD has provided consultation to Accera, AC Immune, Allon, Avanir, AZ Therapies, Banner Health, Biote, Cardeus, Chiesi, Genzyme, GlaxoSmithKline, Hoffman-LaRoche, Janssen, Medivation Inc., Merck and Co. Inc., Nutricia, Pfizer, QR Pharma, Shire, Sonexa, Suven, Takeda, Targacept, Toyama, Transition, and Zinfandel. Her institution has received grant/contract support for clinical trials from Ananir, Genentech, Janssen, and Pfizer. She holds stock options in QR Pharma, Sonexa, and Transition. MF has served as a paid consultant for Accera, Alltech, Astellas, Bayer, Bristol-Myers Squibb, Eisai Medical Research, GE Healthcare, Helicon, Medavante, Medivation Inc., Merck and Co. Inc., Novartis Pharma, Pfizer, Prana Biotech, QR Pharma, Sanofi-Aventis Groupe, Schering-Plough, Lilly, Shire Pharmaceuticals, and Toyama; is a paid speaker for Eisai, Forest, Novartis, and Pfizer; and receives research support from Eisai, Eli Lilly and Co., Genentech, Novartis Pharm., and Roche. MS has served as a consultant/advisor to Amerisciences, BMS, Takeda, Bayer, Eisai, and Pfizer; he receives royalties from Wiley and Amerisciences, and has received contract/grant support from Avid, Lilly, Bayer, Baxter, GE, Pfizer, Janssen, BMS, and Eisai. LL serves as a statistician consultant to Eisai Inc. JM is an employee of Pfizer Inc. RF is an employee of Eisai Inc.

## Authors' contributions

SF, JC, DC, RD, MF, MS, JM and RF helped conceive/design the described analyses, participated in the data analysis, and assisted in the drafting, editing, and interpretation of the manuscript. LL statistically assisted in the planning and performance of the post hoc analyses and assisted in the drafting, editing, and interpretation of the manuscript. All authors read and approved the final version of the manuscript.
